# Investigation of the Cementing Efficiency of Fly Ash Activated by Microsilica in Low-Cement Concrete

**DOI:** 10.3390/ma16216859

**Published:** 2023-10-25

**Authors:** Leonid Dvorkin, Vadim Zhitkovsky, Svetlana Lapovskaya, Yuri Ribakov

**Affiliations:** 1 Institute of Civil Engineering and Architecture, National University of Water and Environmental Engineering, 33028 Rivne, Ukraine; l.i.dvorkin@nuwm.edu.ua (L.D.); v.v.zhitkovsky@nuwm.edu.ua (V.Z.); 2 Ukrainian Research and Design Institute of Building Materials and Products, 04080 Kyiv, Ukraine; labbmsp@ukr.net; 3 Department of Civil Engineering, Ariel University, Ariel 40700, Israel

**Keywords:** low-cement concrete, additives, activation, cementing efficiency, criterion, experimental–statistical models, calculation

## Abstract

This paper presents experimental results on the influence of concrete composition factors on the criterion characterizing the ratio between the compressive strength of activated low-cement concrete and clinker consumption. The investigation was carried out using mathematical planning of the experiments. Experimental and statistical models describing the influence of the fly ash, activating additive (microsilica), consumption of cement and aggregates, as well as the superplasticizer on the strength of low-cement concrete under normal hardening conditions and after steaming were obtained. The values of the clinker efficiency criterion and the mineral additive cementing efficiency coefficient were calculated, and models of these parameters were obtained for the investigated concrete compositions. It was shown that the activating effect of microsilica yields an increase in ash cementing efficiency and clinker efficiency criterion in concrete. Using the obtained models, an example for calculating the ash cementing efficiency coefficient is given.

## 1. Introduction

An important indicator of cement concrete’s economic and environmental efficiency is the ratio between the strength it achieves (*f_cm_*), the specific consumption of Portland cement (C), and accordingly, the clinker component included in it. Both the costs of the main material, the energy resources, and the environmental impact of cement production are associated with the clinker. Increasing the value of criterion L = *f_cm_*/C is especially important for low-cement concrete, the technology for which is attracting more and more attention of researchers and manufacturers [1,2,3]. According to the available data [1], the average value of this criterion for normal strength concrete is 0.1 MPa/(kg/m^3^). For high-strength concrete, it can reach 0.2 or more.

The main way to increase the criterion L in modern concrete technology is by using active mineral additives in combination with superplasticizers [4,5]. The main qualitative indicator of mineral additives is their pozzolanic activity due to the increased reactivity of their constituent silicate and aluminosilicate compounds. The pozzolanic activity of additives varies over a wide range, from 50–100 mg of CaO/g for fly ash to 350–400 and more for highly active materials as microsilica and metakaolin [6,7]. Due to the pozzolanic activity, mineral additives increase the content of hydrated compounds in the cement paste, which has a positive effect on its strength. At the same time, it is important to prevent the possible negative effect of increasing the concrete mixture’s water demand, which is especially characteristic when using highly active and at the same time highly dispersed mineral additives, such as microsilica and metakaolin. This problem is solved by adding to concrete mixtures modern plasticizing additives—superplasticizers.

As active mineral additives in concrete, a large group of dispersed and mainly industrial byproducts is used [8]. Of these, fly ash has found the widest application. Having a high specific surface, comparable to Portland cement, fly ash practically does not increase the water demand of concrete. Fly ash can even slightly reduce the water demand, as it has a certain plasticizing effect due to the vitrified surface of the particles. Adding fly ash limits concrete mixture bleeding and helps retain its workability, increases corrosion resistance, reduces shrinkage deformations, and improves a number of other concrete properties [9,10,11,12]. It is possible to obtain ash–cement concrete with minimum cement and ash consumptions of 150 kg/m^3^ and up to 200 kg/m^3^, respectively. Table 1 shows our data on the value of the strength indicators and criterion L compositions [13] obtained using the ash of the Ladyzhenskaya Thermal Power Plant (Ukraine) for normal weight concrete with a concrete mix slump of 40–50 mm and Portland cement class 32.5. The values were obtained by testing standard 100 mm cube specimens prepared according to [14].

Analysis of the data presented in Table 1 shows that with a cement consumption of 150 kg/m^3^ in a wide range of ash consumption, the value of criterion L both at normal curing and after steaming remains relatively low. To improve the physical and mechanical properties of low-cement fly ash concrete and the criterion L, well-known activation methods developed mainly for cement should be used [15]. These methods, however, are characterized by high energy consumption and require special equipment. More affordable is adding into ash–cement concrete mixtures activating surfactant additives and ultradispersed powders with high pozzolanic activity. A necessary condition for the effectiveness of surfactants is their ability to chemisorb on the surface of mineral additives. In general, cation-active surfactants are recommended for acid-type mineral additives and anion-active ones for base-type [16].

It is recommended to design compositions of low-cement activated concrete using experimental methods [17]. Of the computational and experimental methods, the method of “modified C/W” can be considered the most promising [18,19,20]. As in ordinary concrete design, this method is based on the dependence of the strength on C/W, but it assumes that in the C/W expression (“modified C/W”), the amount of additive that replaces cement without reducing the concrete strength should be taken into account:

(1) $${\left(C/W\right)}_{m}=\frac{C+K_{c.e}\;D}{W},$$

where *C* is the cement content; *D* is the consumption of an active mineral additive; *W* is the water demand; *K_c.e_* is a coefficient considering the mineral additive efficiency.

If necessary, the influence of air introduced into the concrete mixture with additives or porous fillers can be taken into account in the denominator.

The coefficient *K_c.e_* was proposed by A. Smith as the “cementing efficiency” coefficient of mineral additives [10]. It is determined based on experimental data for normal weight concrete as follows:

(2) $$K_{c.e}=\frac{C_{1}-C_{2}}{D}\;,$$

where *C*_1_ is the cement consumption in concrete without additives; *C*_2_ is the cement consumption in concrete with additives without a change in the concrete’s strength.

For concretes of equal strength with the same workability, the saving of cement Δ*C* due to using an active mineral additive (filler) can be found from the equation:

(3) $$\Delta C=K_{c.e}D-{\left(C/W\right)}_{m}\Delta W,$$

where Δ*W* = *W*_0_ − *W_D_* is the change in the concrete mixture water demand when adding additives. *W*_0_ and *W_D_* are the concrete mixture water content without and with the mineral additives, respectively. At Δ*W* < 0, the additive effect decreases, and at Δ*W* > 0, it increases. At $\Delta W>K_{c.e}D$, using active mineral additives does not allow achieving an economy of the cement and clinker or even leads to their overconsumption. This condition determines the advantage of the joint introduction of active additives and plasticizers into the concrete mixture.

The value of *K_c.e._* for an active mineral additive allows finding (*C/W*)*_m_* and designing the concrete composition containing an active mineral additive with a given hardened concrete strength. The value of *K_c.e_* is usually found experimentally and depends on the cement consumption and the additive content. Table 2 presents our experimental data for concrete using fly ash, Portland cements CEM I 32.5 and CEM I 42.5, medium fineness sand and crushed granite stone 5–20 mm, steamed according to the 2 + 3 + 6 + 2 mode at 80 °C [13].

In the present study, statistical models of criterion L for low-cement ash-containing concrete and, based on them, the calculated values of *K_c.e_* for complex mineral additives are presented and analyzed. The novelty of this approach is that the models enable assessment of the main technological factors and evaluation of the effectiveness of adding active components into low-cement concrete containing mineral additives.

## 2. Materials and Methods

Cement–ash concrete was made using Portland cement CEM I (Zdolbuniv, Ukraine) with compressive strength class 32.5, strength after two days–13 MPa, and strength after 28 days–44.5 MPa. Fly ash from Ladyzhyn TPP (Ukraine) was used as the additive in concrete mixtures. The chemical composition of the Portland cement and ash is given in Table 3.

The ash had a specific surface of 310 m^2^/kg, and the pozzolanic activity after 7 days was 15 mg/g, after 28 days was 45 mg/g, and after 60 days was 65 mg/g. To increase the activity of the fly ash, a microsilica additive was used. The microsilica contained 93% ultradispersed amorphous SiO_2_; it was characterized by a specific surface area of 19,000 m^2^/kg and a pozzolanic activity of 420 mg/g.

To solve the problems in the frame of this study, mathematical experiment planning methods [21] were used. Experiments were performed, and after statistical processing of the experimental data, mathematical models were obtained. These models take into account the influence of the cement–ash binders’ composition and additives on the compressive strength and criterion L. The models make it possible to estimate the influence of each of the factors and their interaction effects on the hardened concrete strength after normal curing, after steaming, and its relation to the cement consumption and to obtain the calculated values of the cementing efficiency coefficient of activated ash in concrete of different strengths, taking into account its composition.

When planning the experiment, the following concrete structure parameters were taken as factors:

$X_{1}=\frac{V_{ms}}{V_{ms}+V_{ash}}$—part of the microsilica activator (*V_ms_*) in the total volume of the active mineral additive (*V_ash_*—volume of fly ash);

$X_{2}=\frac{V_{ms}+V_{ash}}{V_{ms}+V_{ash}+V_{c}}$—part of the active mineral additive volume in the total volume of the binder (*V_c_*—cement volume);

$X_{3}=\frac{V_{ms}+V_{ash}+V_{c}}{V_{ms}+V_{ash}+V_{c}+V_{w}}$—part of the binder volume in the binder paste volume (*V_w_*—water volume);

$X_{4}=\frac{V_{ms}+V_{ash}+V_{c}+V_{w}}{V_{ms}+V_{ash}+V_{c}+V_{w}+V_{a}}$—part of the binder paste volume in the concrete volume (*V_a_*—aggregate volume);

$X_{5}=\frac{V_{sp}}{V_{sp}+V_{w}}$—part of the superplasticizer volume in an aqueous solution (*V_sp_*—volume of naphthalene–formaldehyde type superplasticizer).

The conditions for experiment planning and the factor variation range are shown in Table 4.

Transition from the concrete structure parameters to the component’s consumption can be easily carried out using equations assuming that the absolute volume of the concrete mixture is equal to the sum of the volumes of its individual components:

(4) $$MS=X_{1}X_{2}X_{3}X_{4}\rho_{ms},$$

(5) $$Ash=(1-X_{1})\;X_{2}X_{3}X_{4}\rho_{ash},$$

(6) $$C=(1-X_{2})\;X_{3}X_{4}\rho_{c},$$

(7) $$W=(1-X_{3})\;X_{4}\rho_{w},$$

(8) $$A=(1-X_{4})\;\rho_{a},$$

(9) $$SP=\frac{W\cdot X_{5}}{1+X_{5}}\cdot \;\rho_{sp},$$

where *MS*, *Ash*, *C*, *W*, *A*, and *SP* are the consumption of the activator (microsilica), ash, Portland cement, water, aggregate, and superplasticizer, respectively, kg/m^3^; ρ is the components density, kg/m^3^.

The experiment planning matrix for the Ha_5_ plan close to D-optimal [21] is given in Table 5. The concrete composition at the experimental points, the compressive strength of concrete specimens (cubes 100 mm in size) at 28 days of normal curing [14,22] and after steaming according to the (2 + 3 + 6 + 2) mode at 80 °C, as well as the calculated values of criterion L and the cementing efficiency coefficient of the mineral additive *K_c.e_*_._, are given in Table 6.

The concrete compositions for the tested specimens were obtained according to the conditions given in Table 4 and the experiment plan (Table 5). The concrete mix components consumptions are calculated using Equations (4)–(9), according to the values of the factors for each experimental point (Table 5) and are shown in Table 6.

## 3. Results

Statistical processing of the experimental results allowed us to obtain mathematical models of the concrete strength after 28 days of normal curing, the strength of the concrete subjected to steaming, the corresponding criteria L, and the cementing efficiency coefficients of the mineral admixture. The obtained models were considered adequate with a confidence level of 95%, which is considered satisfactory for concrete technology [21]. The models were obtained in the form of polynomial regression equations:

− Concrete compressive strength after 28 days of normal curing

(10) $$\begin{array}{l} f_{cm}^{n.c}=23.4+4.9X_{1}+2.8X_{2}+4.1X_{3}+2.7X_{5}-2.7X_{1}^{2}-2.1X_{2}^{2}-0.3X_{3}^{2}-\\ -0.8X_{5}^{2}+1.9X_{1}X_{5}-0.8X_{1}X_{2} \end{array};$$

− Concrete compressive strength after steaming

(11) $$\begin{array}{l} f_{cm}^{st}=18.5+4.5X_{1}+3.3X_{2}+4.3X_{3}+2.8X_{5}-2.4X_{1}^{2}-1.9X_{2}^{2}-0.5X_{3}^{2}-\\ -0.7X_{5}^{2}+2,4X_{1}X_{5}-0,6X_{1}X_{2} \end{array};$$

− Criterion L for concrete compressive strength after 28 days of normal curing

(12) $$\begin{array}{l} L_{}^{n.c}=0.164+0.035X_{1}+0.045X_{2}+0.013X_{3}+0.02X_{5}-0.02X_{1}^{2}-0.005X_{2}^{2}-0.003X_{3}^{2}+\\ +0.006X_{5}^{2}+0.014X_{1}X_{5}-0.005X_{1}X_{3}-0.004X_{2}X_{5} \end{array}$$

− Criterion L for concrete compressive strength after steaming

(13) $$\begin{array}{l} L_{}^{st}=0.129+0.032X_{1}+0.043X_{2}+0.018X_{3}+0.02X_{5}-0.017X_{1}^{2}-0.005X_{2}^{2}-0.006X_{3}^{2}+\\ +0.005X_{5}^{2}+0.017X_{1}X_{5}-0.004X_{1}X_{3}-0.004X_{2}X_{5} \end{array}$$

− Cementing efficiency coefficient *K_c.e_* for concrete compressive strength after 28 days of normal curing

(14) $$\begin{array}{l} K_{c.e}^{n.c}=0.234+0.13X_{1}+0.2X_{2}-0.09X_{3}+0.07X_{5}-0.07X_{1}^{2}-0.076X_{2}^{2}-0.005X_{3}^{2}-\\ -0.02X_{5}^{2}+0.035X_{1}X_{2}+0.031X_{1}X_{3}+0.05X_{1}X_{5} \end{array}$$

− Cementing efficiency coefficient *K_c.e_* for concrete compressive strength after steaming

(15) $$\begin{array}{l} K_{c.e}^{st}=0.11+0.12X_{1}+0.22X_{2}-0.06X_{3}+0.074X_{5}-0.062X_{1}^{2}-0.073X_{2}^{2}-0.016X_{3}^{2}-\\ -0.02X_{5}^{2}-0.029X_{1}X_{2}-0.029X_{1}X_{3}+0.063X_{1}X_{5} \end{array}$$

**Figure 1:**
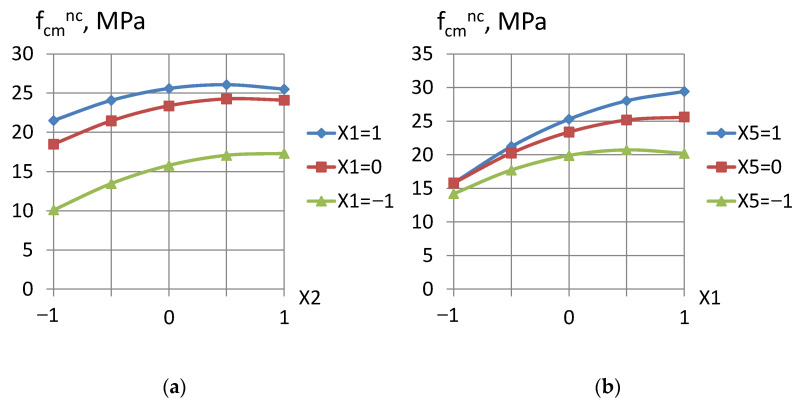
Dependence of the concrete strength at normal curing on (**a**) the active mineral additive part in the binder and (**b**) the part of the activator in the active mineral additive.

Analysis of the obtained models shows that the concrete strength varied in the range from 4.1 to 33.1 MPa for normal curing and from 0.2 to 29.7 MPa after steaming. The transition of all the studied factors’ values from the lower to the upper level helped to increase the concrete strength. The highest impact on the increasing strength was factor *X*_1_ (part of the activator (microsilica) in the volume of mineral additive). A lower effect was caused by factor *X*_2_ (part of the mineral additive in the binder) (Figure 1a). Factor *X*_5_, which characterizes the superplasticizer content, had a quite noticeable influence (Figure 1b). Factor *X*_4_ characterizes the cement paste content in the total volume of concrete. When changing within the specified range, this factor had virtually no effect on the strength characteristics. According to A.M. Neville [23], the ratio between aggregate and cement is a secondary factor for concrete strength, especially for medium and low concrete classes; however, it has been found that constant W/C lean concrete mixtures have higher strength. It is suggested that this trend is due to the water absorption by the aggregate: more aggregate absorbs more water, and the effective water–cement ratio decreases.

There was a significant interaction of factors *X*_1_ and *X*_5_ in the models (Equations (10) and (11)), which is evident in Figure 1b. This interaction showed an increase in activator effectiveness when the superplasticizer content in concrete increases. An increase in the superplasticizer content neutralizes the negative effect of the activator’s (microsilica) large specific surface area on the concrete mixture’s water demand and, accordingly, causes a positive effect on the strength.

**Figure 2:**
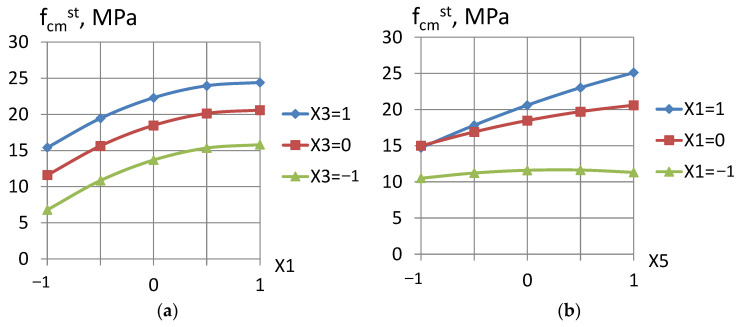
Dependence of the concrete strength after steaming on: (**a**) the activator part in the active mineral additive and (**b**) the part of superplasticizer in the aqueous solution.

The investigated factors affected the concrete strength after steaming in a similar way (Figure 2). Thus, the mineral additive (fly ash) activation, due to addition of microsilica in presence of superplasticizer caused a twofold increase in the concrete strength at normal curing and 2.5-fold at steaming. When using the activator, the cement–ash binder hydration degree increased significantly (by 20–25%) due to the high pozzolanic activity of the microsilica. The structure-forming role of activated fillers is not limited to their significant effect on the hydration degree [24]. Microsilica increases the surface energy of the ash additive, which is manifested by a change in the autocohesion effect and wetting heat [13]. Microsilica increases the strength of the coagulation structure and improves the crystallization conditions of products during the cement–ash hardening, reduces the water and mortar separation of concrete mixtures, and increases the cement adhesive capacity [1].

**Figure 3:**
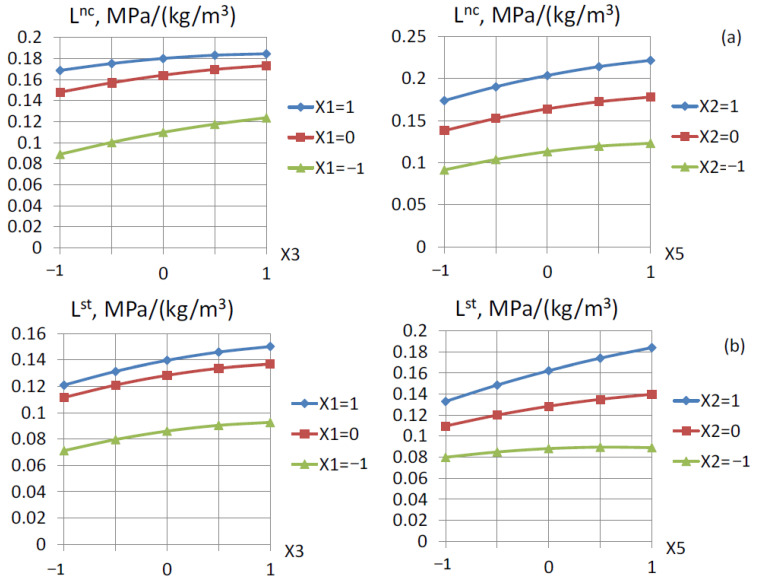
Dependence of criterion L on the investigated factors: (**a**) after normal hardening, (**b**) after steaming.

Based on the experimentally obtained values of the strength at normal curing and after steaming, the values of L as the efficiency criterion for the use of cement and its main component, clinker, and the cementing efficiency coefficient of the mineral additive were calculated at all points of the experiment. Based on these values, mathematical models of criterion L and the coefficient of cementing efficiency for activated fly ash were obtained. Following the obtained mathematical models of criterion L (Equations (12) and (13)), this criterion varied from 0.03 to 0.26 MPa/kg at normal curing (Figure 3a) and from 0.1 to 0.22 at steaming (Figure 3b).

All the investigated factors had a positive effect on criterion L, similar to the effect on the concrete strength. Accordingly, the maximum value of the criterion was observed at the maximum content of the activator (microsilica), fly ash, and superplasticizer.

Table 7 shows the values of the strength and criterion L calculated by the models (Equations (10) and (11)) for the typical experimental compositions of concrete mixtures. As shown in the table, adding the optimal amount of microsilica allows increasing the clinker efficiency by 1.2–2 times.

**Figure 4:**
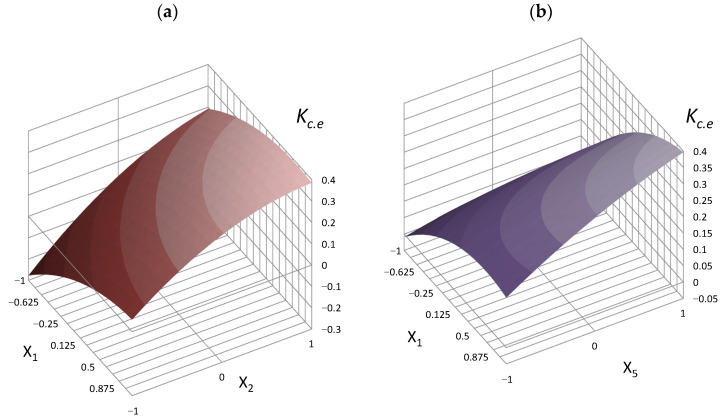
Response surfaces of the cementing efficiency coefficient (*K_c.e_*) of activated fly ash at concrete normal curing vs. the interaction of factors: (**a**) *X*_1_–*X*_2_, (**b**) *X*_1_–*X*_5_.

Analysis of the mathematical models of the active mineral additive cementing efficiency coefficient shows that this indicator depends on the conditions of concrete hardening and varies from −0.36 to 0.61 for concrete after normal curing and from −0.45 to 0.5 for concrete after steaming. An increase in the fly ash cementing efficiency is observed when the content of activator and superplasticizer in the concrete mixture increases. The cementing efficiency coefficient decreases with the cement content increase in concrete. With a high cement content, the addition of activated ash becomes ineffective, and *K_c.e_* acquires negative values (Figure 4). Reducing the cement content, as well as increasing the effective action of the mineral additive due to its activation and the superplasticizer introduction, contributes to an increase in the values of *K_c.e_* and transfers it from the area of negative values to positive ones.

**Figure 5:**
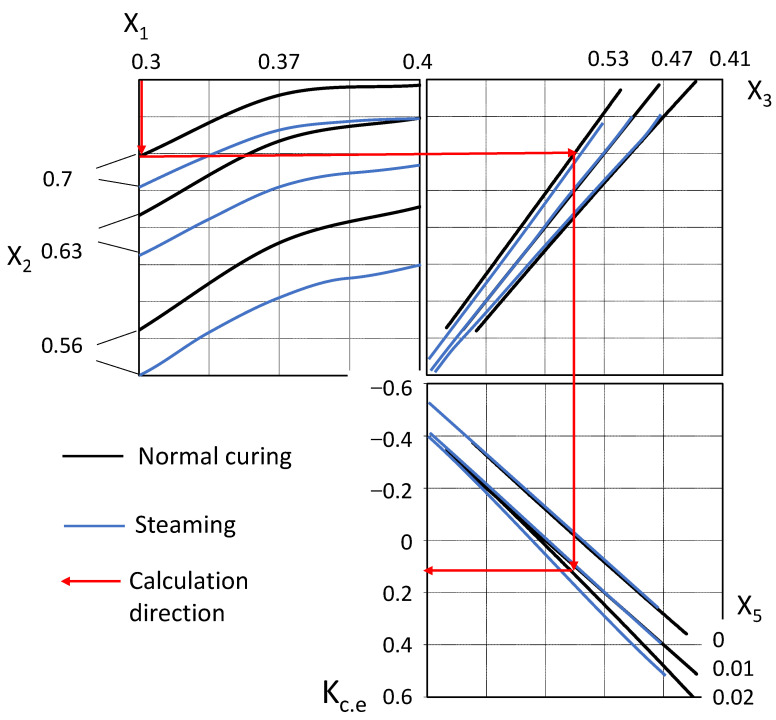
Nomogram for obtaining the cementing efficiency coefficient according to Equations (14) and (15).

For a known consumption of the active mineral additive, cement, and superplasticizer, it is possible to find the cementing efficiency coefficient for the mineral additive using the obtained experimental and statistical models (14–15). For a more convenient graphical solution of the mathematical models (Equations (14) and (15)), a *K_c.e_* nomogram was obtained (Figure 5). According to this nomogram, for the known concrete composition indicators, it is possible to find the value of the active mineral additive cementing efficiency coefficient and use it to calculate the concrete composition with given properties.

Example. Calculate the cementing efficiency coefficient of fly ash when used as an activator of microsilica, if the concrete with activated fly ash has after 28 days of hardening a compressive strength of 21 MPa. Concrete composition of (per 1 m^3^): cement (*C*)—123 kg; water (*W*)—118 L; ash—135 kg; microsilica (*MS*)—70 kg; sand (*S*)—648 kg; crushed stone (*CS*)—1377 kg; superplasticizer (*SP*)—2.6 kg. Density of concrete components: cement—ρ_c_ = 3100 kg/m^3^, ash—ρ_Ash_ = 2600 kg/m^3^; microsilica—ρ_ms_ = 2500 kg/m^3^, superplasticizer—1100 kg/m^3^, water—1000 kg/m^3^.

**Solution.**

1. The part of the activator (microsilica) in the volume of the active mineral additive (*X*_1_) is:

$$X_{1}=\frac{V_{ms}}{V_{ms}+V_{ash}}=\frac{\frac{MS}{\rho_{ms}}}{\frac{MS}{\rho_{ms}}+\frac{Ash}{\rho_{Ash}}}=\frac{\frac{70}{2500}}{\frac{70}{2500}+\frac{135}{2600}}=0.3.$$

2. The part of the active mineral additive in the binder volume (*X*_2_) is:

$$X_{2}=\frac{V_{ms}+V_{ash}}{V_{ms}+V_{ash}+V_{c}}=\frac{\frac{MS}{\rho_{ms}}+\frac{Ash}{\rho_{Ash}}}{\frac{MS}{\rho_{ms}}+\frac{Ash}{\rho_{Ash}}+\frac{C}{\rho_{c}}}=\frac{\frac{70}{2500}+\frac{135}{2600}}{\frac{70}{2500}+\frac{135}{2600}+\frac{123}{3100}}=0.7.$$

3. The part of the binder in the binding paste volume (*X*_3_) is:

$$X_{3}=\frac{V_{ms}+V_{ash}+V_{c}}{V_{ms}+V_{ash}+V_{c}+V_{w}}=\frac{\frac{MS}{\rho_{ms}}+\frac{Ash}{\rho_{Ash}}+\frac{C}{\rho_{c}}}{\frac{MS}{\rho_{ms}}+\frac{Ash}{\rho_{Ash}}+\frac{C}{\rho_{c}}+\frac{W}{\rho_{w}}}=\frac{\frac{70}{2500}+\frac{135}{2600}+\frac{123}{3100}}{\frac{70}{2500}+\frac{135}{2600}+\frac{123}{3100}+\frac{118}{1000}}=0.53$$

4. The part of the superplasticizer in the aqueous solution (*X*_5_) is:

$$X_{5}=\frac{V_{sp}}{V_{sp}+V_{w}}=\frac{\frac{SP}{\rho_{sp}}}{\frac{SP}{\rho_{sp}}+\frac{W}{\rho_{w}}}=\frac{\frac{2.6}{1100}}{\frac{2.6}{1100}+\frac{118}{1000}}=0.02.$$

5. From Equation (14) or according to the nomogram (Figure 5), the value of *K_c.e_* is:

*K_c.e_* = 0.11.

## 4. Conclusions

A set of experimental–statistical dependences of strength, criterion L, and cementing efficiency coefficient at normal hardening and steaming was obtained for cement–ash concrete with microsilica additive and a naphthalene–formaldehyde-type superplasticizer using the mathematical experiment planning method.

The analysis of the obtained models made it possible to establish the range of changes of the studied parameters and their influence on the concrete composition factors. It has been found that activation by mineral additive (fly ash), due to the addition of microsilica in presence of a superplasticizer yields a twofold increase in the concrete strength at normal hardening, and after steaming the strength increases about 2.5 times.

The compositions of cement–ash concrete with a cement consumption of 100–140 kg/m^3^ exhibited the maximum efficiency criterion L; these compositions contained the maximum microsilica and superplasticizer values.

To find the fly ash cementing efficiency coefficient values, a corresponding nomogram was created. It allowed taking into account the influence of the cement, ash, activating mineral, and superplasticizer consumption and the concrete hardening conditions.

The obtained cementing efficiency coefficient can be used to calculate compositions of concrete containing fly ash.

## Figures and Tables

**Table 1:** The value of criterion L for cement–ash concretes with cement and ash consumptions of 150 kg/m^3^ and 50–200 kg/m^3^, respectively.

Consumption, kg/m^3^		Binder/Water	Compressive Strength, MPa			Criterion L		
Cement (C)	Ash (A)		After Steaming *f_cm_′*	28 Days after Steaming *f_cm_″*	After 28 Days of Normal Curing *f_cm_*‴	*f_cm_′* C	*f_cm_″* C	*f_cm_‴* C
150	-	0.89	4.4	6.4	7.8	0.029	0.043	0.052
150	50	1.15	9.7	11.6	11.4	0.065	0.077	0.076
150	75	1.28	11.6	14.0	10.6	0.077	0.093	0.072
150	100	1.42	14.2	14.9	12.5	0.095	0.099	0.083
150	125	1.58	14.4	16.1	15.6	0.096	0.107	0.104
150	150	1.72	15.7	16.6	14.7	0.105	0.110	0.098
150	175	1.85	15.6	16.8	15.3	0.104	0.112	0.102
150	200	2.0	16.1	17.3	16.6	0.107	0.115	0.111

**Table 2:** Experimental data on *K_c.e_* for ash-containing concretes.

Concrete Class by Compression Strength	Cement Class	Ash Consumption, kg/m^3^	*C*/*W*	(*C*/*W*)*_m_*	Cement Consumption, kg/m^3^	*K_c.e_*
Normal curing						
C12/15	32.5	-	1.46	-	293	-
		150	1.23	1.46	246	0.31
C15/20	32.5	-	1.79	-	357	-
		150	1.58	1.79	317	0.27
C20/25	42.5	-	1.66	-	331	-
		150	1.49	1.66	298	0.22
Steaming						
C12/15	32.5	-	1.46	-	293	-
		200	1.16	1.46	233	0.3
C15/20	32.5	-	1.79	-	357	-
		200	1.53	1.79	305	0.26
C20/25	42.5	-	1.66	-	331	-
		200	1.46	1.66	291	0.2

**Table 3:** Chemical composition of the Portland cement and fly ash (%).

Material	CaO	SiO_2_	Al_2_O_3_	Fe_2_O_3_ + FeO	MgO	Na_2_O + K_2_O	SO_3_	L.O.I. *
Portland cement	64.4	21.3	5.7	3.5	0.8	0.9	2.5	0.4
Fly ash	2.3	55.4	26.6	8.5	1.6	4.4	0.3	0.5

* L.O.I.—loss on ignition.

**Table 4:** Experiment planning conditions.

Variation Factors	Factors’ Variation Levels		
	−1	0	+1
*X*_1_	0.3	0.37	0.44
*X*_2_	0.56	0.63	0.7
*X*_3_	0.41	0.47	0.53
*X*_4_	0.25	0.265	0.28
*X*_5_	0.0	0.01	0.02

**Table 5:** Experiment planning matrix (Ha_5_).

Experiment Point No.	Factors Value in Coded Form				
	*X*_1_	*X*_2_	*X*_3_	*X*_4_	*X*_5_
1	+1	+1	+1	+1	+1
2	−1	−1	+1	+1	+1
3	−1	+1	−1	−1	−1
4	+1	−1	−1	−1	−1
5	−1	+1	−1	+1	+1
6	+1	−1	−1	+1	+1
7	+1	+1	+1	−1	−1
8	−1	−1	+1	−1	−1
9	−1	+1	+1	+1	−1
10	+1	−1	+1	+1	−1
11	+1	+1	−1	−1	+1
12	−1	−1	−1	−1	+1
13	−1	+1	+1	−1	+1
14	+1	−1	+1	−1	+1
15	+1	+1	−1	+1	−1
16	−1	−1	−1	+1	−1
17	+1	0	0	0	0
18	−1	0	0	0	0
19	0	+1	0	0	0
20	0	−1	0	0	0
21	0	0	+1	0	0
22	0	0	−1	0	0
23	0	0	0	+1	0
24	0	0	0	−1	0
25	0	0	0	0	+1
26	0	0	0	0	−1
27	0	0	0	0	0

**Table 6:** Concrete compositions at experimental points, values of strength, and calculated values of criterion L and coefficient *K_c.e_*.

No.	Consumption of Concrete Components, kg/m^3^						Compressive Strength, MPa		Criterion L,		Cementing Efficiency Coefficient *K_c.e_*	
	*MS*	*Ash*	*C*	*W*	*A*	*SP*	*f_cm_* (nc)	*f_cm_* (st)	nc	st	nc	st
1	114	151	138	132	1944	3.0	33.1	29.7	0.240	0.215	0.32	0.26
2	62	151	202	132	1944	3.0	13.9	9.3	0.069	0.046	−0.33	−0.43
3	54	131	95	148	2025	0.0	11.3	7.7	0.119	0.081	0.21	0.11
4	63	84	140	148	2025	0.0	11.7	5.3	0.084	0.038	−0.02	−0.26
5	60	146	107	165	1944	3.7	12.9	8.5	0.121	0.080	0.26	0.13
6	71	94	157	165	1944	3.7	20.9	15.7	0.133	0.100	0.31	0.12
7	102	135	123	118	2025	0.0	23.9	19.3	0.194	0.157	0.16	0.08
8	56	135	181	118	2025	0.0	12.3	8.5	0.068	0.047	−0.36	−0.45
9	78	189	138	132	1944	0.0	19.5	16.3	0.141	0.118	0.08	0.02
10	91	121	202	132	1944	0.0	19.9	13.9	0.098	0.069	−0.19	−0.33
11	79	104	95	148	2025	3.3	24.9	21.1	0.261	0.221	0.61	0.50
12	43	104	140	148	2025	3.3	5.7	0.7	0.041	0.005	−0.24	−0.42
13	70	169	123	118	2025	2.6	21.1	17.1	0.171	0.139	0.11	0.04
14	82	108	181	118	2025	2.6	29.1	24.3	0.161	0.134	0.01	−0.10
15	88	117	107	165	1944	0.0	15.7	10.7	0.147	0.100	0.34	0.20
16	48	117	157	165	1944	0.0	4.1	0.2	0.026	0.100	−0.30	−0.45
17	86	114	143	140	1985	1.6	25.6	20.6	0.179	0.144	0.29	0.16
18	59	143	143	140	1985	1.6	15.8	11.6	0.111	0.081	0.04	−0.07
19	81	143	116	140	1985	1.6	24.1	19.9	0.208	0.172	0.35	0.25
20	65	114	170	140	1985	1.6	18.5	13.3	0.109	0.078	−0.03	−0.18
21	82	145	161	125	1985	1.4	27.2	22.3	0.169	0.138	0.11	0.01
22	63	112	125	156	1985	1.7	19	13.7	0.152	0.110	0.35	0.18
23	77	136	151	148	1944	1.6	23.4	18.5	0.155	0.123	0.23	0.11
24	68	121	135	133	2025	1.5	23.4	18.5	0.174	0.137	0.23	0.11
25	73	129	143	140	1985	3.2	25.3	20.6	0.177	0.144	0.28	0.16
26	73	129	143	140	1985	0.0	19.9	15	0.139	0.105	0.14	0.02
27	73	129	143	140	1985	1.6	23.4	18.5	0.164	0.129	0.23	0.11

Notes: art of sand in aggregate (sand and crushed stone)–0.32; nc—normal curing (28 days), st—steaming.

**Table 7:** Calculated values of the concrete strength and criterion L.

Structure Parameters				Materials’ Consumption, kg/m^3^				Compressive Strength, MPa, at 28 Days	Criterion L
*X*_1_	*X*_2_	*X*_3_	*X*_5_	*MS*	*Ash*	*C*	*W*		
Microsilica activator									
0.3	0.70	0.53	0.02	63	148	128	122	22	0.172
0.37	0.70	0.53	0.02	78	133	128	122	30	0.234
0.3	0.70	0.41	0.02	48	112	99	153	15	0.152
0.37	0.70	0.41	0.02	60	103	99	153	19	0.192
0.44	0.70	0.53	0.02	93	119	128	122	33	0.258
0.37	0.63	0.47	0.02	62	107	140	138	23	0.164
Without activator									
0.0	0.47	0.36	0.0	-	113	174	189	15	0.086
0.0	0.47	0.36	0.02	-	113	174	164	20	0.115

Note: Concrete compositions with *MS* additive were calculated at *X*_4_ = 0.26.

## Data Availability

Not applicable.
